# Fish responses to underwater sounds depend on auditory adaptations: An experimental test of the effect of motorboat sounds on the fish community of a large fluvial lake

**DOI:** 10.1002/ece3.10946

**Published:** 2024-03-11

**Authors:** Jérôme Barbeau, Renata Mazzei, Marco A. Rodríguez, Raphaël Proulx

**Affiliations:** ^1^ Research Centre for Watershed—Aquatic Ecosystem Interactions Université du Québec à Trois‐Rivières Trois‐Rivières Quebec Canada

**Keywords:** anthropogenic sounds, auditory structures, avoidance behavior, ecoacoustics, fish, freshwater, underwater soundscape

## Abstract

Freshwater fishes exhibit a wide range of auditory adaptations and capabilities, which are assumed to help them navigate their environment, avoid predators, and find potential mates. Yet, we know very little about how freshwater environments sound to fish, or how fish with different auditory adaptations respond to different soundscapes. We first compiled data on fish hearing acuity and adaptations and provided a portrait of how anthropogenic sounds compare to natural sounds in different freshwater soundscapes. We then conducted a sound‐enrichment field experiment at Lake Saint Pierre, a large fluvial lake in Canada, to evaluate the effect of motorboat sound exposure on the fish community by looking at the extent to which changes in species abundances were linked to auditory adaptations. Data compilation showed that the hearing acuity of most species overlaps with a wide range of ambient and anthropogenic underwater sounds while the field experiment showed that species with more specialized auditory structures were captured less often in sound‐enriched traps, indicating avoidance behavior. Our findings highlight the importance of considering species' sensorial adaptations when evaluating the community‐scale effects of anthropogenic sounds on the fish community, especially at low levels of anthropogenic activity.

## Graphical abstract

Freshwater fishes exhibit a wide range of auditory adaptations and capabilities. We found that the hearing acuity of most species overlapped with a wide range of ambient and anthropogenic underwater sounds, and species with more specialized auditory structures were captured less often in sound‐enriched traps, indicating avoidance behavior.

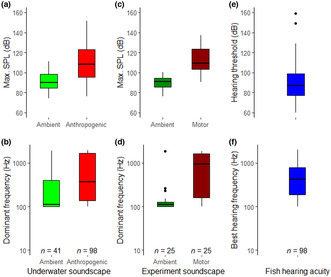

## 1 INTRODUCTION

Sounds produced by human activities in the aquatic environment, such as motorized transport, dredging, seismic exploration, or installation of underwater structures, have the potential to impact a range of aquatic organisms (McQueen et al., 2020; Popper et al., 2020; Shannon et al., 2016). Merchant ships, in particular, are known to negatively affect the behavior of organisms across taxonomic groups in the marine environment, including large mammals (Chion et al., 2017; Richardson et al., 2013) and fish (Handegard et al., 2003; Picciulin et al., 2010; Vabø et al., 2002; Whitfield & Becker, 2014). Our knowledge of the effects of anthropogenic sounds on marine fish has significantly progressed in the last few decades (reviewed by Carroll et al., 2017; Di Franco et al., 2020; Popper et al., 2019, 2020). Nonetheless, there is still a considerable amount of work ahead in order to attain an equivalent depth of knowledge regarding freshwater fish (Cox et al., 2018; Mickle & Higgs, 2017; Pieniazek et al., 2020; Popper & Hawkins, 2019; Roca et al., 2018).

One of the challenges of studying anthropogenic sounds in freshwater environments is that the underwater soundscape is very complex and diverse. Shallow depths, combined with complex bathymetry, types of substrates, and hydrodynamics, make the soundscape particularly heterogeneous (Kuehne et al., 2013; Proulx et al., 2019). For example, the underwater soundscape of a river changes abruptly in sound intensity and spectral signature over short distances and depending on the flow regime (Kacem et al., 2020; Tonolla et al., 2010, 2011; Wysocki et al., 2007). Furthermore, the soundscape is modified by anthropogenic sounds originating from different sources, such as recreational motorboats and shoreline activities (Whitfield & Becker, 2014; Wysocki et al., 2007). A portrait of the underwater soundscape across 173 locations (i.e., lakes, ponds, and rivers) showed that sounds produced by motorboats and aquatic organisms overlap in their peak frequencies (Rountree et al., 2020). The same study identified that sounds produced by organisms are less frequent when the acoustic background gets increasingly louder. Therefore, to investigate the impact of anthropogenic sounds on freshwater organisms, we need more studies that combine soundscape characterization with in situ experimental tests.

The response of organisms to the underwater soundscape will depend on their sensitivity to sounds. Fish is one of the most diverse groups of vertebrates, and besides exhibiting a great diversity of morphologies and behaviors, they have evolved a wide range of inner ear and specialized auditory structures (Ladich, 2013; Ladich & Schulz‐Mirbach, 2016; Popper et al., 2003). For instance, two‐thirds of freshwater fish species have a direct connection between the swim bladder and inner ear via a chain of specialized ossicles, the Weberian apparatus (Nakatani et al., 2011), which suggests that hearing plays an important role in this group. The inner ear, the primary hearing organ in fish and other vertebrates, displays a broad range of variation in the anatomy of otoliths, sensory epithelia, and sensory hair cells across taxa (Ladich, 2013; Ladich & Schulz‐Mirbach, 2016; Popper et al., 2003). Fish also exhibit a diversity of gas‐filled cavities, such as the swim bladder, which primarily serve to regulate buoyancy, but can also improve hearing by transmitting sounds to the inner ear (Ladich & Schulz‐Mirbach, 2016; Wiernicki et al., 2020). Yet, the selective forces leading to the evolution of such specializations are still poorly understood, as is how the presence of auditory structures influences fish responses to underwater sounds (Kunc et al., 2016; Ladich & Schulz‐Mirbach, 2016; Popper & Hawkins, 2019).

One of the anticipated responses of fish subjected to underwater anthropogenic sounds is avoidance. Experiments conducted in captive fish (Fewtrell & McCauley, 2012; Murchy et al., 2017; Neo et al., 2015; Pearson et al., 1992) and free‐moving fish (Brehmer et al., 2019; Handegard et al., 2003; Picciulin et al., 2010; Vabø et al., 2002; Wheeland & Rose, 2015) suggest that avoidance of motorboat sounds is not systematic and is likely related to phylogenetic affiliation. A recent sound‐enrichment experiment with motorboat sounds carried out in the Detroit River (Great Lakes region) found that fish in the family Cyprinidae (which have specialized auditory structures) showed avoidance behavior, whereas those in the families Gobiidae, Centrarchidae, and Percidae (which do not have specialized auditory structures) were indifferent to the sound enrichment (Pieniazek et al., 2020). These results suggest that fish species with auditory adaptations may be more likely to avoid noise pollution, even at low levels of sound intensity.

In this study, we first present a compilation of hearing acuity data in fish in relation to the presence of accessory auditory structures. We then produce a portrait of different types of underwater soundscapes to represent the extent of acoustic ambiances and anthropogenic events in lakes and rivers. Finally, we present the results of a sound‐enrichment experiment conducted in the littoral zone of a large fluvial lake in Quebec, Canada, which aims to understand how fish with different auditory adaptations respond to anthropogenic sounds. We predicted that fish species with more specialized auditory structures have better hearing acuity and are more likely to avoid motorboat sounds.

## 2 MATERIALS AND METHODS

### 2.1 Auditory acuity data

We compiled hearing acuity data from all complete fish audiograms published in the literature, where both hearing thresholds and best hearing frequency were available (Ladich & Fay, 2013; Mann et al., 2007; Nedwell et al., 2004). When raw data were not directly available, we used the software WebPlotDigitizer (Rohatgi, 2021) to extract values from plots. We extracted the lowest hearing thresholds (dB re 1 μPa), that is, the lowest sound pressure level (SPL) in the audiogram, and the best hearing frequency (Hz), that is, the frequency at the lowest hearing threshold, for 98 freshwater species. We classified species into one of the following categories of auditory structures (Ladich & Schulz‐Mirbach, 2016): the presence of swim bladder extensions to the inner ear (Swim bladder extension; *n* = 2); the presence of a direct connection between the swim bladder and the inner ear via a chain of ossicles (Weberian apparatus; *n* = 46); the presence of air‐filled cavities near the inner ear (air‐filled cavities, *n* = 13); the presence of a swim bladder without connection to the inner ear (only swim bladder, *n* = 31) and the absence of a swim bladder (no swim bladder, *n* = 6).

### 2.2 Underwater soundscape

We recorded underwater sounds from June to October 2019 (ice‐free period) to unveil the soundscape of a wide range of freshwater environments under ambient (*n* = 41) and anthropogenic (*n* = 96) conditions (see Supporting Information, and Tables S1 and S2 for locations, habitats, and anthropogenic activities). From the shore or a boat, we plunged a piezoelectric hydrophone to a depth of 0.5 to 1 m to record audio sequences of 5 to 20 s. We sampled anthropogenic sounds in the presence of human activities, such as the passing of recreational boats and merchant ships, the use of fishing lures, bathing and swimming, and port activities. Ambient sounds were recorded after the disturbance had stopped and the acoustic environment returned to its natural state, usually a few minutes later. We discarded sequences with interference caused by the sampling procedure (e.g., friction on the hydrophone probe caused by dragging vegetation, or waves hitting the boat).

We recorded all audio sequences in 16‐bit WAV format at a rate of 44.1 kHz using a portable digital device (H2n Handy Recorder, Zoom, Tokyo, Japan) connected to an H1 hydrophone and a PA4 amplifier (Aquarian Scientific, Anacortes, USA). The recording system had a maximum input of 10 V (rms), a gain of 20 dB, and a sensitivity of −190 dB re 1 V/μPa over the frequency range of 10–3000 Hz (Kacem et al., 2020; Roca et al., 2018). We obtained the SPL, referenced in dB re 1 μPa, with the PAMGuide functions (Merchant et al., 2015). We calculated the root mean square (SPL _RMS_ dB re. 1 μPa) power spectrum by 5 Hz intervals over the frequency range 100–2000 Hz using a 50% overlap Hann window. For each sequence, we recovered the maximum SPL (dB) and the dominant frequency (Hz) of the power spectrum. The maximum SPL corresponds to the highest sound amplitude over the frequency range, whereas the dominant frequency is the interval associated with the max SPL.

### 2.3 Sound‐enrichment experiment

#### 2.3.1 Study site

The experiment was conducted at Lake Saint Pierre (LSP), Quebec, Canada (46°12′7.42″ N; −72°49′22.18″ W), from June to October 2020 (see Figure S2 for a map with locations). LSP is a widening of the St. Lawrence River, approximately 40 km long and 14 km wide, located between the towns of Sorel‐Tracy and Trois‐Rivières. It is a shallow ecosystem with an average depth of 2.7 m (De La Chelenière et al., 2015). The lake harbors a highly diverse fish community of ~78 species (Benoît et al., 1987) and is an area of conservation interest, designated as a biosphere reserve by UNESCO in 2000. Besides its ecological relevance, LSP is of great socio‐economic importance. It serves as a passage for merchant ships circulating in the seaway—a pathway that permits oceangoing vessels to travel from the Atlantic Ocean to the Great Lakes. It also has a busy traffic of recreational boats, being a favorite spot for fishing and nautical activities. These activities are a substantial source of underwater anthropogenic sounds in the system.

#### 2.3.2 Experimental design

We used passive traps to explore the effect of sound enrichment on the fish community of LSP (Figure S1). Traps were deployed at shallow (1.5 to 2 m deep) sites with slow flow, near aquatic grass beds. We used fyke nets, with two 15 m wings that directed fish toward a retention cage 1.2 m high by 1.8 m wide. These traps were deployed on the bottom of the lake to capture fish passively once they had moved through the mouth and funnels and entered the retention cage.

Each experimental trial consisted of two traps deployed simultaneously, at least 200 m apart from each other. One trap was used as control and the other for the sound‐enrichment treatment. The sound‐enriched treatment had an aluminum boat with a running engine anchored 2 m behind the retention cage so that approaching fish could not see the boat (average Secchi depth in summertime at LSP is <1.2 m). The engine used for sound enrichment was a four‐stroke outboard 9.9 HP Yamaha, which is commonly used by boaters in LSP. Experimental trials were paired as follows: During the first experimental trial, traps were set at control and treatment locations and the engine would run continuously, in the water, with the propeller turning at minimum thrust for 3 h at the treatment location. The boat was secured with three anchors to ensure it remained in position and water circulation was oriented to the opposite side of the traps, to reduce turbulence toward the traps. At the end of the 3‐h period, the fish present in the retention cages of both the control and the treatment traps were identified into species and counted. During the second trial, the boat was positioned behind the trap previously used as control, and empty traps were redeployed at the same locations for another 3 h (i.e., the treatment and control locations were switched). After the engine had again run continuously for 3 h, the fish captured in both retention cages were identified and counted. Fish counting in the two cages took about 30 min and live fish were always released offshore. This procedure was repeated over 15 days of fishing (60 trials in total, 30 with sound enrichment and 30 without sound enrichment) in different areas of LSP (Figure S2). Experiments were conducted at different periods of the day: early morning (*n* = 10), afternoon (*n* = 14), evening (*n* = 24), and early night (*n* = 12). This study protocol was approved by the Animal Care Committee of the University of Quebec at Trois‐Rivières (certificate #2020‐RP1).

In parallel with the sound‐enrichment experiment, we deployed hydrophones at the entrance of the trap to characterize underwater sounds during the experiments. We retrieved and analyzed the audio sequences following the procedure described in the Section 3.2.

### 2.4 Statistical analyses

We used the hierarchical modeling of species communities (HMSC) approach (Ovaskainen & Abrego, 2020; Ovaskainen et al., 2017) to examine fish species' responses to the increase in sound levels induced experimentally by the boat motor. Statistically, the HMSC is a multivariate generalized mixed model that represents the joint response of all species to the covariates used in the model. In addition to treatment or environmental covariates, the model can integrate features of the sampling design, which may introduce dependence among samples, effects of species traits of their responses to the covariates, and potential dependence in species responses resulting from phylogenetic relationships. Species counts were assumed to have a Poisson distribution whose mean was modeled on the log scale as a function of a linear predictor comprising a covariate term and two random effect terms, one for spatial location and another for the blocks representing treatment‐control paired locations in the experimental design. The covariate in the linear predictor was the sound‐enrichment treatment coded as a binary factor. The species' responses to the sound‐enrichment treatment (i.e., the regression coefficients associated with species covariates) were, in turn, modeled as a function of a single species trait, the presence of a hearing adaptation, coded as a binary factor. We obtained a species phylogeny using packages ape (Paradis et al., 2004) and fishtree (Chang et al., 2019) in R (Figure S3), and used the phylogenetic correlation model of Ives and Helmus (2011) to allow for potential dependence among species' responses. The model allowed us to assess whether the abundance of species with particular hearing adaptations differed between the control and experimental treatments after accounting for potential dependence among samples induced by the sampling design or by a phylogenetic signal.

Model fitting was performed in a Bayesian framework using the Hmsc package (Tikhonov et al., 2020). We used the default prior distributions in the package. Posterior distributions for the model parameters were obtained using the Markov chain Monte Carlo (MCMC) algorithm implemented in Hmsc. A single MCMC chain was run for 500,000 iterations; the first 100,000 were treated as a warm‐up phase and were discarded and the remaining iterations were thinned (1:100) to yield a sample of 4000 posterior distribution values. Convergence of the chain was assessed by the Gelman‐Rubin criterion implemented in the rstan package in R (Stan Development Team, 2022).

## 3 RESULTS

### 3.1 Fish auditory acuity

**FIGURE 1:**
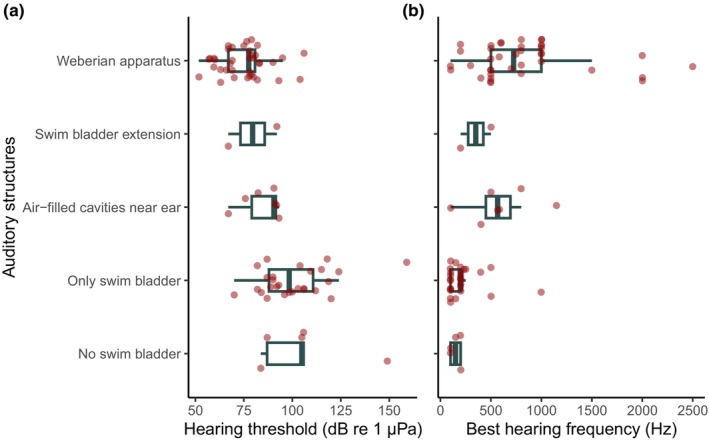
Average hearing threshold (a) and best hearing frequency (b) in 98 freshwater fish species (compiled from Ladich & Fay, 2013, Mann et al., 2007, Nedwell et al., 2004) with different hearing structures. The central line in boxes, the boxes, and the whiskers indicate the median, Q1, Q3, and 1.5*QR. Dots represent individual data points.

The literature review of fish audiograms revealed that the presence of specialized auditory structures was associated with a decrease in hearing threshold and an increase in the best hearing frequency (Figure 1). The hearing threshold decreased from a median of 102 dB in fishes without any auditory adaptation to 76.8 dB in fishes with a Weberian apparatus (Figure 1a) while species in other categories had intermediate values (only swim bladder: 96 dB; air‐filled cavities near ear: 90.5 dB; swim bladder extension: 89.1 dB). Furthermore, species with the Weberian apparatus had higher sensitive frequencies (median: 725 Hz) than freshwater species in the other categories (no swim bladder: 200 Hz; only swim bladder: 200 Hz; air‐filled cavities near ear: 567 Hz; swim bladder extension: 365 Hz, Figure 1b).

### 3.2 Underwater soundscape

**FIGURE 2:**
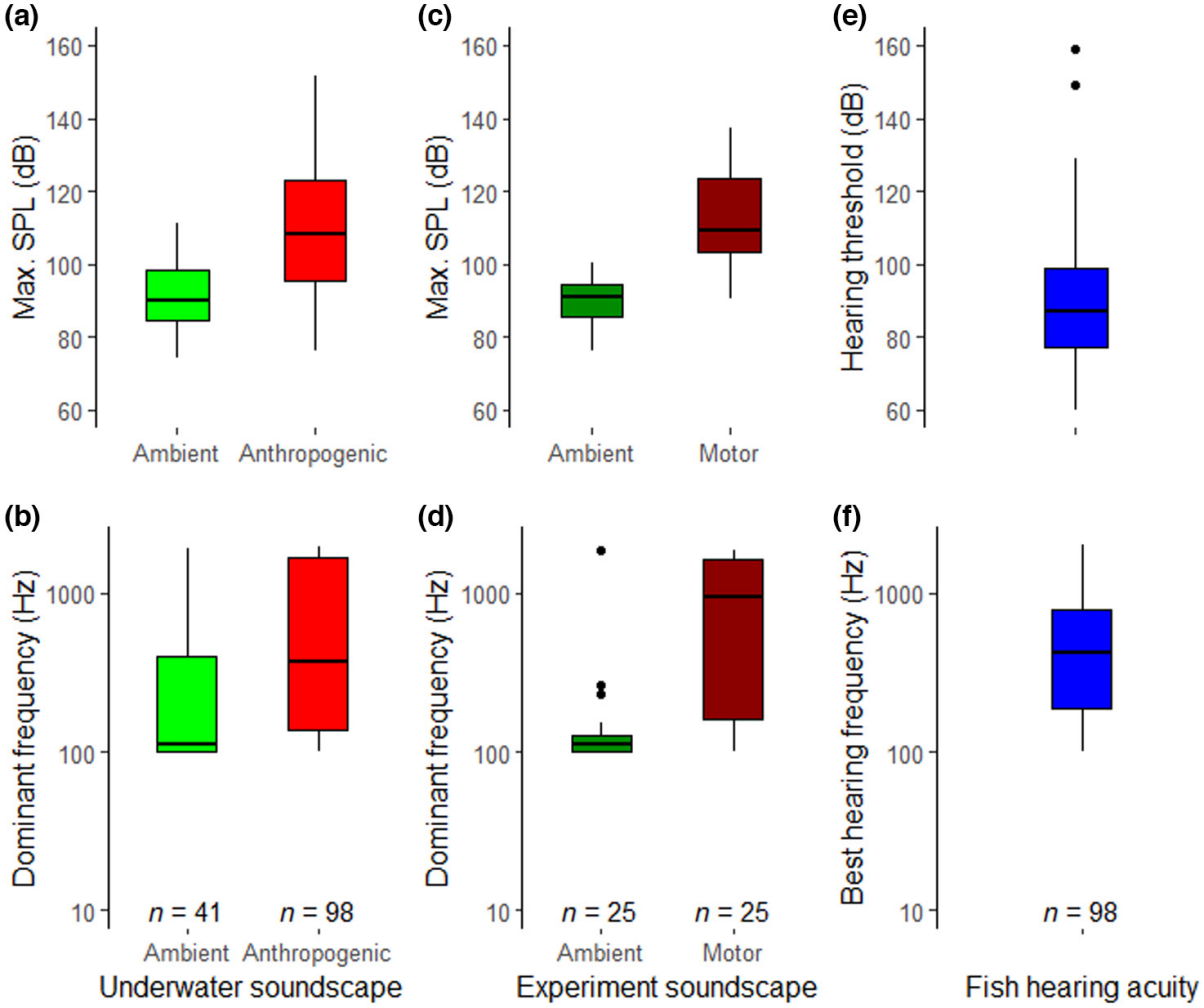
Boxplots showing freshwater fish hearing acuity, types of underwater sound sources recorded in lakes and rivers, and sound‐enrichment treatments of the LSP experiment: Panels (a) and (b) show the maximum sound pressure level (max SPL) and the dominant frequency for underwater recordings of ambient (*n* = 41) and anthropogenic (*n* = 96) sounds. Panels (c) and (d) show the same two variables (max SPL and dominant frequency) for underwater recordings at the entrance of the trap in the sound‐enrichment experiments that included two treatments: ambient (control) or motor sounds. Panels (e) and (f) report the lowest hearing threshold and the best hearing frequency of freshwater fishes from published audiograms (compiled from Ladich & Fay, 2013, Mann et al., 2007, Nedwell et al., 2004). The central line in boxes, the boxes, and the whiskers indicate the median, Q1, Q3, and 1.5*QR. Dots indicate outliers.

Anthropogenic events recorded across different water bodies were louder and dominated by higher‐frequency sounds (Figure 2), adding on average 18 dB to the ambient soundscape (Figure 2a). The maximum sound pressure level (max SPL) recorded was 152 dB for anthropogenic sounds and 111 dB for ambient sounds (Figure 2a). Dominant frequencies of underwater recordings ranged from 100 to 2000 Hz (Figure 2b). Ambient sounds showed frequencies with a median of 111 Hz while anthropogenic sounds consisted of frequencies with a median of 370 Hz (Figure 2b).

The sound‐enrichment experiment conducted in LSP successfully increased the underwater sounds at the entrance of the treatment traps. The sound‐enrichment treatment increased the max SPL by an average of 22 dB (Figure 2c). LSP was characterized by ambient sounds of low dominant frequency, with a median of 110 Hz, while the motorboat treatment generated sounds of higher frequencies at the entrance of the trap, with a median of 956 Hz (Figure 2d).

The sounds recorded during the experiment were similar to the ambient and anthropogenic sounds recorded in other freshwater environments. The max SPL of most anthropogenic events recorded in lakes and rivers was above the lowest hearing threshold of 75% of the freshwater fish species listed in this study (Figure 2e) and the dominant frequencies of anthropogenic events fully overlapped with the best hearing frequencies of the listed species (Figure 2f).

### 3.3 Sound‐enrichment experiment

We captured a total of 26,272 fish belonging to 30 species (Table S3). Of these fish, 19,235 had a Weberian apparatus (15 species), 25 had a swim bladder extension (one species), and 7012 did not have a swim bladder or a connection between the swim bladder and inner ear (14 species, grouped and reclassified as “without accessory hearing structures”). Species with air‐filled cavities close to the inner ear do not naturally occur in LSP.

**FIGURE 3:**
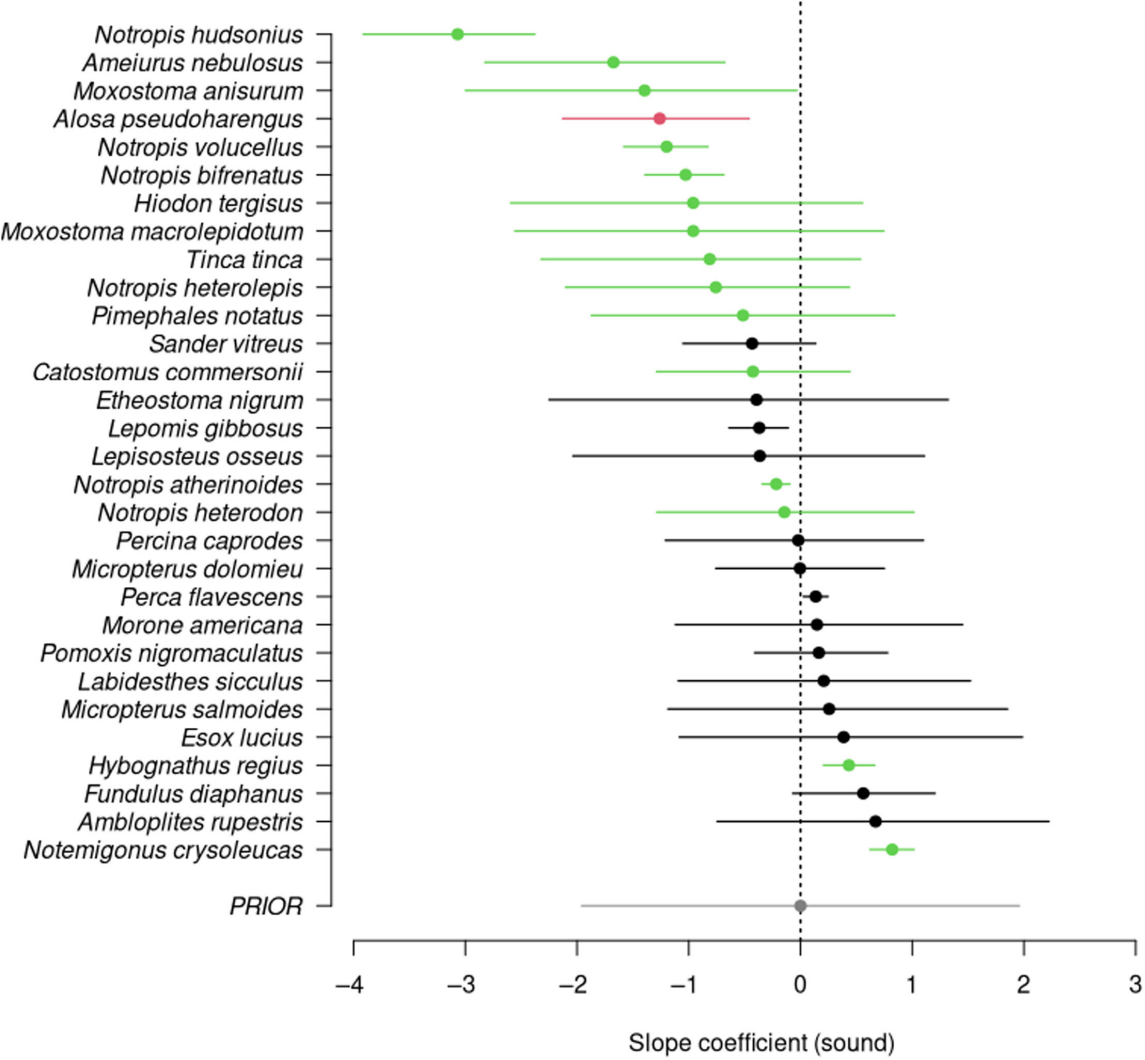
Summary of the posterior distribution of regression coefficients reflecting the effect of the sound‐enrichment treatment on catches (means and 95% credible intervals). Positive or negative values indicate that species were captured more or less often in sound‐enriched traps, respectively. Credible intervals crossing zero indicate lower confidence in the effect estimate. Colors refer to species with no particular auditory specialization (black), species with a connection between the inner ear and swim bladder (green), and a single species with an extension from the swim bladder to the inner ear (red). The prior distribution summary for the regression coefficients is also shown (gray).

The HSMC analysis considering all species simultaneously indicated an overall negative effect of the presence of specialized auditory structures (Weberian apparatus or swim bladder extension), suggesting avoidance of the sound‐enrichment treatment (Table S3, effect size median: −0.77; 95% credible interval: −1.51, 0.03, Figure S4). When looking at the effect by species, the analysis revealed that 14 of 16 species with more specialized auditory structures had negative coefficient values (Table S3; Figure 3). The effect was particularly contrasted in six species (see Table S3 for coefficients per species), including four of the genus *Notropis* (Cypriniformes): *Alosa pseudoharengus*, *Ameiurus nebulosus*, *Notropis bifrenatus*, *Notropis atherinoides*, *Notropis hudsonius*, and *Notropis volucellus*. Three species of the family Catostomidae (Cypriniformes) had negative coefficient values: *Catostomus commersonii*, *Moxostoma anisurum*, and *Maxostoma macrolepidotum*. Brown bullhead (*Ameiurus nebulosus*), the sole representative of the order Siluriformes, also showed a negative coefficient.

Among species without specialized auditory structures, five were associated with a negative coefficient and nine with a positive coefficient, suggesting no systematic trends in response to sound enrichment (Figure 3). A weak positive effect of the sound‐enrichment treatment was detected for only one species without specialized auditory structure: pumpkinseed (*Lepomis gibbosus*). Interestingly, the other sunfish species caught in the experiment (*Ambloplites rupestris*, rock sunfish) had a coefficient of the opposite sign, indicating that closely related species may show contrasting responses to motorboat sound enrichment.

## 4 DISCUSSION

We investigated the potential effect of underwater anthropogenic sounds on the behavior of freshwater species with different auditory adaptations. We first compiled, from published audiograms, variables describing the hearing acuity of different fish species and compared them to the levels of ambient and anthropogenic sounds recorded in a variety of freshwater environments. We then conducted a field experiment to evaluate the influence of motorboat sounds on the behavior of 30 fish species. Our results can be summarized as three important findings: first, species with more specialized auditory structures have a higher auditory acuity than species with more basic structures. Second, variables characterizing the hearing acuity of most species overlapped with a wide range of ambient and anthropogenic underwater sounds recorded in freshwater environments. Finally, our experiment showed that species with specialized auditory structures, like the Weberian apparatus or swim bladder elongation toward the inner ear, avoided sound‐enriched traps.

### 4.1 Hearing acuity and underwater soundscape

Our compilation of fish audiogram data illustrated the great variation that exists in hearing thresholds (from 45 to 159 dB) and best hearing frequencies (from 20 to 3000 Hz) across species. Our recordings of the underwater soundscape under different conditions revealed that the max SPL of ambient sounds was typically close to the hearing threshold of fish species compiled here, but that the dominant frequency of the same sounds was below their best hearing frequency. These results suggest that the ambient soundscape is a rather “quiet” environment for a majority of freshwater fish. Furthermore, they also underline how little we know about the evolutionary forces that shaped differences in hearing acuity among fish. In this regard, the eco‐acoustical constraints hypothesis states that the hearing threshold and frequency bandwidth of fish have co‐evolved with the acoustic background of water bodies (Ladich, 2013). In contrast, the auditory scene analysis proposes that the auditory system of fish involves multiple functions that aim at enhancing the detection and segregation of specific sound sources in the environment (Fay & Popper, 2000). Neither of the above hypotheses has been thoroughly tested so far on a large number of species. Fundamental questions, such as why fish have such good hearing or why is there so much variation in their hearing sensitivity, still require a proper answer.

Our data compilation also showed that the presence of specialized auditory structures, such as the Weberian apparatus, was associated with lower hearing thresholds and increased best hearing frequencies. However, results of audiogram testing should be interpreted with caution due to the presence of multiple sources of error. Yet, another recent review exploring the relationship between auditory specializations and sound frequency detection in fish came to similar conclusions with regard to best hearing frequency (Wiernicki et al., 2020). In particular, they found a positive correlation between best frequency and maximum frequency, suggesting a larger hearing bandwidth in auditory specialists, irrespective of phylogeny or type of audiogram test (Wiernicki et al., 2020). Furthermore, another set of audiogram data compiled on 87 marine fish species revealed similar trends for both hearing threshold and best hearing frequency across the same specialization categories (unpublished). Overall, our results support the proposition that in general fish equipped with specialized auditory structures should be more sensitive to anthropogenic sounds as previously suggested elsewhere (table 2 in Popper & Hawkins, 2019).

### 4.2 Sound‐enrichment experiment

Our experiment indicates that species with more specialized auditory structures are more likely to avoid anthropogenic sounds, as these species were captured less in traps exposed to motor sounds. In particular, four species of the genus *Notropis* (Cypriniformes: Cyprinidae) avoided sound‐enriched traps, with *Notropis hudsonius* being the species with the strongest negative coefficient. A recent experiment using playbacks of motor sounds in the natural environment also revealed a strong avoidance response in *Notropis hudsonius* (Pieniazek et al., 2020).

The order Cypriniformes is phylogenetically grouped with Siluriformes and Clupeiformes in the clade Otocephala, which includes fish with a mechanical link between the swim bladder and the inner ear. Among the 30 species investigated in the present study, the brown bullhead (*Ameiurus nebulosus*) was the only species of the order Siluriformes, while the alewife (*Alosa pseudoharengus*) was the only representative of the order Clupeiformes. The family Catostomidae, in the Cypriniformes, was represented by three species: *Maxostoma anisurum*, *Maxostoma macrolepidotum*, and *Catostomus commersonii*. The five Otocephala species in the present study had a tendency to avoid traps subject to sound enrichment, further supporting the role of the Weberian apparatus on anthropogenic sound avoidance.

Fish species without specialized auditory structures did not avoid motor sounds, as they were equally likely to be caught in traps with or without enrichment. These included some species of importance for recreational fishing, such as the yellow perch (*Perca flavescens*; *n* = 6496 total captures), pumpkinseed (*Lepomis gibbosus*; *n* = 329), walleye (genus *Sander*; *n* = 59), and bass (genus *Micropterus*; *n* = 25). Our compilation of fish audiograms for the above genera reveals that hearing thresholds are above 85 dB at best hearing frequencies around 100 Hz. These are typical values for fish with a swim bladder but no connection to the inner ear. By comparison, the average sound level (max SPL) and dominant frequency at the entrance of sound‐enriched traps were 109 dB and 956 Hz, respectively. Thus, it is likely that most of the energy associated with the motor sounds in the experiment was above the best hearing frequency of species without specialized auditory structures. This would explain why they did not avoid the sound‐enriched traps. Similar results were reported by McCormick et al. (2019), which showed that engines with different acoustic signatures had contrasting effects on the behavioral response of different fish species.

It is important to emphasize that anthropogenic sounds recorded in our study do not reflect the characteristics of the sound at its source. The sounds we recorded correspond to different acoustic events to which fish in lakes and rivers are commonly exposed in the presence of anthropogenic activities. For reference, exposure limits for sounds that cause fish mortality and injury correspond to an increase of more than 100 dB above ambient values (e.g., Popper & Hawkins, 2019). Therefore, the fact that many species avoided the motor sounds in our experiment indicates that even low levels of anthropogenic sound pollution may induce a behavioral response. This is particularly important considering the wide range of boats and engines commonly used in LSP, which could influence fish communities to different degrees, depending on the combination of sound characteristics and species' hearing sensitivities.

The bioacoustics literature regularly refers to two mechanisms of sound detection in fish: pressure wave detection via the vibration of an air cavity such as the swim bladder that is then transmitted to the inner ear, and mechanical wave detection via the movement of otoliths in response to particle acceleration (Popper et al., 2003; Popper & Hawkins, 2019). It is generally accepted that fish with a connection between the swim bladder and the inner ear can perceive sounds via both modes of hearing, whereas fish without a connection mainly detect particle acceleration. These differences would partly explain the lower hearing thresholds and the higher sensitive frequencies in species with a connection. However, we did not measure particle acceleration in our study, and thus max SPL levels reported here should not be interpreted as measures of exposure criteria to sound pollution. The relative contribution of sound wave propagation modes can differ greatly near the sound source and depends in particular on the size of the propeller, the sound wavelength, the water depth, and the type of substrate (Schellart & Popper, 1992). More studies are required to corroborate our results on the impact of underwater anthropogenic sounds in other freshwater environments and fish species.

Studies using remote monitoring methods, such as telemetry or echo sounding, have reported avoidance behavior in fish when a motorized boat is approaching (Handegard et al., 2003; Jacobsen et al., 2014; Vabø et al., 2002). These methods generally target one or two species and the authors acknowledge that it is sometimes difficult to separate acoustic effects from visual stimuli (e.g., approaching boat, change in turbidity). These same methods, however, have the advantage of working with actual motorboat sounds rather than sounds replayed through a loudspeaker. It has also been noted that the effect of sounds on fish behavior fades over time, suggesting habituation (Harding et al., 2018; Nedelec et al., 2015). Our sound‐enrichment experiment sought to minimize several of these potential biases. First, the fish had to swim actively toward the entrance of the trap and the sound source before being caught. The motorboat was located well behind the trap, reducing its potential to become a visual cue. Our experiment represented a baseline situation in terms of sound exposure, as the motor sounds were produced at minimum thrust. Fish in LSP are often exposed to motor sounds much louder than those used in our experiment, implying that fish were not naive to this type of sound pollution. Finally, we studied 30 fish species with different auditory adaptations, which allowed us to address the effect of sound enrichment at the community level.

## 5 CONCLUSIONS

The impact of underwater sound pollution is a growing conservation issue, as avoidance responses by aquatic organisms can lead to changes in habitat use. In Quebec, provincial fishing regulations identify exclusion zones for some species during the spawning season. The precautionary principle (Kriebel et al., 2001) would call for restricting the presence of motorboats over these areas during this key period of the life cycle. The present study suggests that it is essential to consider the presence of auditory adaptations to better understand and predict the behavioral response of fish to underwater sound pollution. Other sensory traits such as the ability to communicate acoustically, or the characteristics of the lateral line could be equally important. Our results also give important insights into the factors driving the evolution of auditory adaptations in fish, as we found that hearing in freshwater fishes is well‐matched with the acoustic environment of freshwater bodies, suggesting that specialized hearing might be advantageous in quieter environments.

## AUTHOR CONTRIBUTIONS

**Jérôme Barbeau:** Conceptualization (supporting); data curation (equal); formal analysis (equal); funding acquisition (supporting); investigation (equal); methodology (supporting); project administration (equal); visualization (equal); writing – original draft (equal); writing – review and editing (equal). **Renata Mazzei:** Data curation (supporting); formal analysis (supporting); validation (equal); visualization (equal); writing – original draft (equal); writing – review and editing (equal). **Marco A. Rodríguez:** Conceptualization (equal); data curation (equal); formal analysis (equal); methodology (equal); project administration (equal); supervision (equal); validation (equal); visualization (equal); writing – original draft (supporting); writing – review and editing (equal). **Raphaël Proulx:** Conceptualization (equal); data curation (equal); formal analysis (equal); funding acquisition (lead); investigation (equal); methodology (equal); project administration (equal); supervision (equal); validation (equal); visualization (equal); writing – original draft (supporting); writing – review and editing (equal).

## CONFLICT OF INTEREST STATEMENT

The authors declare no conflict of interest.

## Supporting information


Data S1

## Data Availability

The data that support the findings of this study are openly available in figshare at http://doi.org/10.6084/m9.figshare.22764599.
