# Supplementary material for: Fish responses to underwater sounds depend on auditory adaptations: An experimental test of the effect of motorboat sounds on the fish community of a large fluvial lake
Source: Ecol Evol. 2024 Mar 11;14(3):e10946. doi: 10.1002/ece3.10946 (PMC10926058; doi:10.1002/ece3.10946)

**Supplementary Materials**

**Table S1**. List of anthropogenic acoustic events (n = 98) recorded using a hydrophone from June to October 2019 in several water bodies in the province of Québec, Canada. The duration of the recordings varied between 5 and 20 seconds. For each event, we noted the hydrophone depth (H. depth) and retrieved the maximum intensity (Max SPL, in db re 1 µpa) and the dominant frequency (Dom. freq., in hz).

| Sound description | Environment | Max SPL | Dom. freq. | Latitude | Longitude | H. depth (m) |
| --- | --- | --- | --- | --- | --- | --- |
| Music on the surface | Lake | 112 | 276 | 46.78175 | -72.87646 | 1 |
| Music on the surface | Lake | 120 | 276 | 46.78175 | -72.87646 | 0.1 |
| Crowded beach | Lake | 105 | 1731 | 46.76609 | -72.91822 | 0.5 |
| Walking with heavy steps | Lake | 114 | 126 | 46.762343 | -72.99184 | 0.5 |
| Walking normally | Lake | 101 | 161 | 46.762343 | -72.99184 | 0.5 |
| Sitting on the dock an tipping the feet in the water | Lake | 96 | 1621 | 46.762343 | -72.99184 | 0.5 |
| Whistling | Lake | 86 | 131 | 46.762343 | -72.99184 | 0.5 |
| Music on the surface | Lake | 89 | 111 | 46.762343 | -72.99184 | 0.5 |
| Music on the surface | Lake | 93 | 1831 | 46.762343 | -72.99184 | 0.5 |
| Music on the surface | Lake | 86 | 116 | 46.762343 | -72.99184 | 0.5 |
| Music on the surface | Lake | 99 | 586 | 46.762343 | -72.99184 | 0.1 |
| Music on the surface | Lake | 99 | 736 | 46.762343 | -72.99184 | 0.1 |
| Music on the surface | Lake | 96 | 106 | 46.762343 | -72.99184 | 0.1 |
| Ricochets of stones | Lake | 85 | 841 | 46.762343 | -72.99184 | 0.5 |
| Music on the surface | Lake | 111 | 271 | 46.762343 | -72.99184 | 0 |
| Music on the surface | Lake | 108 | 291 | 46.762343 | -72.99184 | 0 |
| Music on the surface | Lake | 108 | 371 | 46.762343 | -72.99184 | 0 |
| 7 people on the dock, 2 rabaskas and 1 kayak | Lake | 91 | 121 | 46.77746 | -72.88696 | 0.2 |
| Canoe and kayak | Lake | 102 | 1901 | 46.753852 | -73.05475 | 0.5 |
| Car driving over a bridge | Lake | 79 | 111 | 46.753852 | -73.05475 | 0.5 |
| Canoe | Lake | 90 | 101 | 46.75775 | -73.06879 | 1 |
| 5 kayaks | Lake | 92 | 421 | 46.76152 | -73.08661 | 1 |
| Pontoon anchored in two places. Boats over 300m. Two people snorkeling. | Lake | 95 | 1586 | 45.10784 | -74.41387 | 1 |
| Sailboat with gasoline engine | Lake | 105 | 101 | 45.10784 | -74.41387 | 1 |
| GP3R fireworks | River | 126 | 1991 | 46.33018 | -72.52965 | 1 |
| GP3R fireworks | River | 129 | 1941 | 46.33018 | -72.52965 | 1 |
| Medium speed pedalo | Lake | 122 | 151 | 47.032548 | -71.42118 | 1 |
| High speed pedalo | Lake | 125 | 181 | 47.032548 | -71.42118 | 1 |
| Marina with a rowboat approaching | River | 106 | 1921 | 46.68125 | -71.87777 | 1 |
| Car traffic on a bridge | River | 98 | 101 | 46.22542 | -72.6226 | 1 |
| 2-stroke 2-force low-speed engine | River | 126 | 101 | 46.22759 | -72.62382 |  |
| 2-stroke 2-stroke engine at full speed | River | 152 | 141 | 46.22759 | -72.62382 |  |
| 200-300m upstream of a hydroelectric dam | River | 111 | 1976 | 46.448822 | -72.74466 | 0.1 |
| Medium speed pedalo | Lake | 120 | 361 | 46.63428 | -72.59992 | 1 |
| Motor 150 forces in neutral | River | 145 | 1891 | 46.351668 | -72.53165 | 1 |
| 2 personal watercraft | River | 116 | 1901 | 46.351668 | -72.53165 | 1 |
| 1 pontoon and 1 personal watercraft and 6 boats in action | River | 127 | 1866 | 46.351668 | -72.53165 | 1 |
| Noise under a bridge | River | 118 | 1716 | 46.357791 | -72.53715 | 1 |
| Motor boat 115 forces at low revs | River | 134 | 211 | 46.357791 | -72.53715 | 1 |
| Noise below deck and personal watercraft | River | 122 | 1266 | 46.366943 | -72.55743 | 1 |
| Crankbait | River | 128 | 1256 | 46.333977 | -72.51493 | 1 |
| Popper surface lure | River | 102 | 1966 | 46.333977 | -72.51493 | 1 |
| Spinnerbait | River | 89 | 136 | 46.333977 | -72.51493 | 1 |
| Frog surface lure | River | 92 | 1726 | 46.333977 | -72.51493 | 1 |
| crankbait | River | 93 | 1866 | 46.333977 | -72.51493 | 1 |
| Propeller crankbait | River | 98 | 1971 | 46.333977 | -72.51493 | 1 |
| Horizontal jig | River | 93 | 106 | 46.333977 | -72.51493 | 1 |
| Buzzbait | River | 102 | 1851 | 46.333977 | -72.51493 | 1 |
| Dare devil spoon | River | 92 | 111 | 46.333977 | -72.51493 | 1 |
| Spinner | River | 89 | 1696 | 46.333977 | -72.51493 | 1 |
| Jigging crankbait | River | 104 | 1956 | 46.333977 | -72.51493 | 1 |
| Vibrato Sebile | River | 97 | 106 | 46.333977 | -72.51493 | 1 |
| Anchor scratching at the bottom | River | 115 | 1736 | 46.366943 | -72.55743 | 1 |
| Movement of people in the boat | River | 104 | 216 | 46.36648 | -72.44856 | 1 |
| Smash with an oar | River | 123 | 206 | 46.36648 | -72.44856 | 1 |
| Moving a gas can | River | 127 | 221 | 46.36648 | -72.44856 | 1 |
| Rowing | River | 102 | 281 | 46.36648 | -72.44856 | 1 |
| Talking | River | 95 | 176 | 46.36648 | -72.44856 | 1 |
| Speaking out loud | River | 105 | 481 | 46.36648 | -72.44856 | 1 |
| Motor 20 forces in neutral | River | 143 | 261 | 46.36648 | -72.44856 | 1 |
| Cars driving on a road | River | 87 | 121 | 46.304215 | -72.5329 | 1 |
| Merchant boat leaving a port | River | 127 | 1581 | 46.31776 | -72.54883 | 1 |
| Diver at 1m | River | 114 | 381 | 45.14798 | -74.35325 | 1 |
| Diver at 10m | River | 108 | 411 | 45.14798 | -74.35325 | 1 |
| Diver at 5m | River | 110 | 371 | 45.14798 | -74.35325 | 1 |
| Boat pulling an inner tube | River | 93 | 1906 | 45.14798 | -74.35325 | 1 |
| Ambient sound with leisure boat in the channel | River | 106 | 1821 | 46.272389 | -72.61964 | 1 |
| Working cottage water pump | Lake | 76 | 136 |  |  |  |
| 50 volume submersible cargo speaker |  | 125 | 426 |  |  |  |
| Canoe and kayak | Lake | 110 | 1286 |  |  |  |
| Really loud boat noise in the distance: with a sailboat engine not far away | River | 126 | 236 |  |  |  |
| Boat pulling an inner tube and pontoon pulling a wakeboard | River | 116 | 361 |  |  |  |
| Passage of a large boat | River | 93 | 131 |  |  |  |
| Passage of a boat | River | 121 | 446 |  |  |  |
| Removing an anchor | River | 104 | 101 |  |  |  |
| Dropping an anchor | River | 116 | 256 |  |  |  |
| Hovercraft | River | 100 | 121 |  |  |  |
| Purse seine (fishing gear) | Pond | 89 | 101 |  |  |  |
| Scaring at 15m 1 person | Pond | 87 | 111 |  |  |  |
| Scaring 20m with 2 people | Pond | 86 | 101 |  |  |  |
| Car driving | River | 82 | 101 |  |  |  |
| Upstream of a hydroelectric dam | River | 144 | 161 |  |  |  |
| Upstream of a hydroelectric dam | River | 146 | 176 |  |  |  |
| Downstream of a hydroelectric dam | River | 112 | 121 |  |  |  |
| Downstream of a hydroelectric dam | River | 119 | 156 |  |  |  |
| 2 personal watercraft | River | 119 | 1826 |  |  |  |
| 1 pontoon and 1 personal watercraft and 6 boats in action | River | 133 | 1001 |  |  |  |
| noise under a bridge | River | 118 | 1556 |  |  |  |
| watercraft | River | 109 | 1806 |  |  |  |
| noise under a bridge | River | 101 | 106 |  |  |  |
| 20 horsepower engine won't start | River | 105 | 1951 |  |  |  |
| merchant ship | River | 126 | 1641 |  |  |  |
| Two merchant ships | River | 128 | 546 |  |  |  |
| Two boats anchored, one with the engines running | River | 130 | 1101 |  |  |  |
| Volga Cargo Boat | River | 129 | 421 |  |  |  |
| Dredger boat Ocean Traverse North | River | 135 | 1706 |  |  |  |
| 1 pontoon, 1 personal watercraft and 6 displacement boats | River | 140 | 1161 |  |  |  |
| 1 pontoon, 1 personal watercraft and 6 boats on the move | River | 139 | 1936 |  |  |  |

**Table S2.** List of ambient acoustic events (n=41) recorded using a hydrophone from June to October 2019 in several water bodies in the province of Québec, Canada. The duration of the recordings varied between 5 and 20 seconds. For each event, we noted the hydrophone depth (H. depth) recovered the maximum intensity (Max SPL, in db re 1 upa) and the dominant frequency (Dom. freq., in hz).

| Sound description | Environment | Max SPL | Dom. freq. | Latitude | Longitude | H. depth (m) |
| --- | --- | --- | --- | --- | --- | --- |
| Ambient sound | Lake | 80 | 101 | 46.78175 | -72.87646 | 1 |
| Ambient sound | Lake | 81 | 101 | 46.762343 | -72.991835 | 0.5 |
| Ambient sound | Lake | 85 | 101 | 46.753852 | -73.054752 | 0.5 |
| Ambient sound | Lake | 76 | 221 | 46.75775 | -73.06879 | 1 |
| Ambient sound | Lake | 87 | 101 | 46.76152 | -73.08661 | 1 |
| Ambient sound | Lake | 106 | 311 | 45.10784 | -74.41387 | 1 |
| Ambient sound | Lake | 84 | 136 | 47.032548 | -71.421177 | 1 |
| Trout jumping on the surface | Lake | 87 | 101 | 47.032899 | -71.421252 | 0.5 |
| Ambient sound | Lake | 94 | 101 | 47.032924 | -71.421389 | 0.2 |
| Ambient sound | Lake | 93 | 101 | 47.033277 | -71.42117 | 0.2 |
| Small basin of a stream | Stream | 90 | 261 | 47.033513 | -71.42092 | 0.1 |
| Small basin of a stream | Stream | 99 | 566 | 47.033513 | -71.42092 | 0.1 |
| Stream | Stream | 93 | 116 | 47.024815 | -71.422377 | 0.1 |
| Stream | Stream | 99 | 176 | 47.024815 | -71.422377 | 0.1 |
| Stream | Stream | 86 | 101 | 47.024815 | -71.422377 | 0.1 |
| Stream under a culvert | Stream | 74 | 101 | 47.024815 | -71.422377 | 0.1 |
| Marina | River | 92 | 1791 | 46.68125 | -71.877766 | 1 |
| Ambient sound | River | 82 | 126 | 46.24032 | -72.64617 | 1 |
| Ambient sound | River | 84 | 106 | 46.2334 | -72.63754 | 1 |
| Moderate rain | River | 110 | 761 | 46.278711 | -72.596302 | 0.5 |
| Ambient sound | River | 85 | 111 | 46.333977 | -72.51493 | 1 |
| Ambient sound | River | 90 | 101 | 46.31236 | -72.53892 | 1 |
| Ambient sound | River | 111 | 1856 | 46.34147 | -72.537231 | 1 |
| Ambient sound | River | 100 | 396 | 45.14798 | -74.35325 | 1 |
| Ambient sound | River | 102 | 106 | 46.33609 | -72.51868 | 1 |
| Ambient sound | River | 109 | 1851 | 46.336289 | -72.517661 | 1 |
| Ambient sound | River | 105 | 1781 | 46.272389 | -72.619635 | 1 |
| Boat waves coming out on a dock | River | 103 | 1926 | 46.272532 | -72.619285 | 1 |
| Ambient sound |  | 77 | 101 |  |  |  |
| Ambient sound |  | 86 | 131 |  |  |  |
| Ambient sound |  | 80 | 101 |  |  |  |
| Ambient sound |  | 83 | 106 |  |  |  |
| Ambient sound |  | 101 | 101 |  |  |  |
| Ambient sound |  | 92 | 106 |  |  |  |
| Ambient sound |  | 90 | 126 |  |  |  |
| Ambient sound |  | 86 | 1266 |  |  |  |
| Ambient sound under a bridge |  | 82 | 111 |  |  |  |
| Thunder | River | 88 | 101 |  |  |  |
| Ambient sound |  | 95 | 1881 |  |  |  |
| Ambient sound |  | 91 | 1846 |  |  |  |
| Ambient sound |  | 90 | 106 |  |  |  |

Table S3. Information table for the species captured during the sound enrichment experiment. See the methods section for a detailed description of how experiments were performed.

| **Order** | **Family** | **Species** | **Adaptation** | **Catches / Model statistics** | | | |
| --- | --- | --- | --- | --- | --- | --- | --- |
|  |  |  |  | Total catches | Model Coefficient | lower 95% CI | upper 95% CI |
| **Atheriniformes** | Atherinopsidae | *Labidesthes sicculus* | No specialization | 5 | 0.2082 | -1.0944 | 1.5215 |
| **Centrarchiformes** | Centrarchidae | *Ambloplites rupestris* | No specialization | 3 | 0.6719 | -0.7465 | 2.2232 |
|  |  | *Lepomis gibbosus* | No specialization | 329 | -0.3689 | -0.6405 | -0.1094 |
|  |  | *Micropterus dolomieu* | No specialization | 24 | -0.0046 | -0.7578 | 0.7503 |
|  |  | *Micropterus salmoides* | No specialization | 1 | 0.2564 | -1.1862 | 1.8527 |
|  |  | *Pomoxis nigromaculatus* | No specialization | 40 | 0.1644 | -0.4111 | 0.7798 |
| **Clupeiformes** | Clupeidae | *Alosa pseudoharengus* | Swimbladder extension | 25 | -1.2602 | -2.1292 | -0.4597 |
| **Cypriniformes** | Catostomidae | *Catostomus commersonii* | Weberian apparatus | 16 | -0.4258 | -1.2884 | 0.4431 |
|  |  | *Moxostoma anisurum* | Weberian apparatus | 4 | -1.3954 | -2.9995 | -0.0311 |
|  |  | *Moxostoma macrolepidotum* | Weberian apparatus | 1 | -0.9604 | -2.5566 | 0.7458 |
| **Cypriniformes** | Leuciscidae | *Hybognathus regius* | Weberian apparatus | 8436 | 0.4329 | 0.2056 | 0.6643 |
|  |  | *Notemigonus crysoleucas* | Weberian apparatus | 1328 | 0.8206 | 0.621 | 1.0165 |
|  |  | *Notropis atherinoides* | Weberian apparatus | 8810 | -0.217 | -0.3442 | -0.094 |
|  |  | *Notropis heterolepis* | Weberian apparatus | 6 | -0.7578 | -2.1028 | 0.438 |
|  |  | *Notropis volucellus* | Weberian apparatus | 215 | -1.1976 | -1.5815 | -0.8286 |
|  |  | *Notropis bifrenatus* | Weberian apparatus | 214 | -1.0279 | -1.3926 | -0.6858 |
|  |  | *Notropis heterodon* | Weberian apparatus | 9 | -0.1444 | -1.2862 | 1.0155 |
|  |  | *Notropis hudsonius* | Weberian apparatus | 165 | -3.0684 | -3.9135 | -2.3783 |
|  |  | *Pimephales notatus* | Weberian apparatus | 4 | -0.5146 | -1.8725 | 0.8432 |
| **Cypriniformes** | Tincidae | *Tinca tinca* | Weberian apparatus | 4 | -0.8121 | -2.3223 | 0.5377 |
| **Cyprinodontiformes** | Fundulidae | *Fundulus diaphanus* | No specialization | 38 | 0.562 | -0.0702 | 1.2034 |
| **Esociformes** | Esocidae | *Esox lucius* | No specialization | 2 | 0.3862 | -1.0846 | 1.9864 |
| **Eupercaria** | Moronidae | *Morone americana* | No specialization | 5 | 0.1476 | -1.1198 | 1.4507 |
| **Hiodontiformes** | Hiodontidae | *Hiodon tergisus* | Weberian apparatus | 1 | -0.9605 | -2.5947 | 0.5568 |
| **Lepisosteiformes** | Lepisosteidae | *Lepisosteus osseus* | No specialization | 1 | -0.3642 | -2.0375 | 1.1089 |
| **Perciformes/**  **Percoidei** | Percidae | *Etheostoma nigrum* | No specialization | 1 | -0.3932 | -2.2501 | 1.3214 |
|  |  | *Perca flavescens* | No specialization | 6496 | 0.1364 | 0.0258 | 0.2466 |
|  |  | *Percina caprodes* | No specialization | 8 | -0.02 | -1.2099 | 1.1007 |
|  |  | *Sander vitreus* | No specialization | 42 | -0.4331 | -1.0525 | 0.1367 |
| **Siluriformes** | Ictaluridae | *Ameiurus nebulosus* | Weberian apparatus | 18 | -1.6745 | -2.8248 | -0.6778 |

**Figure S1.** Schematic drawing of the passive traps used in the sound-enrichment experiment.

**
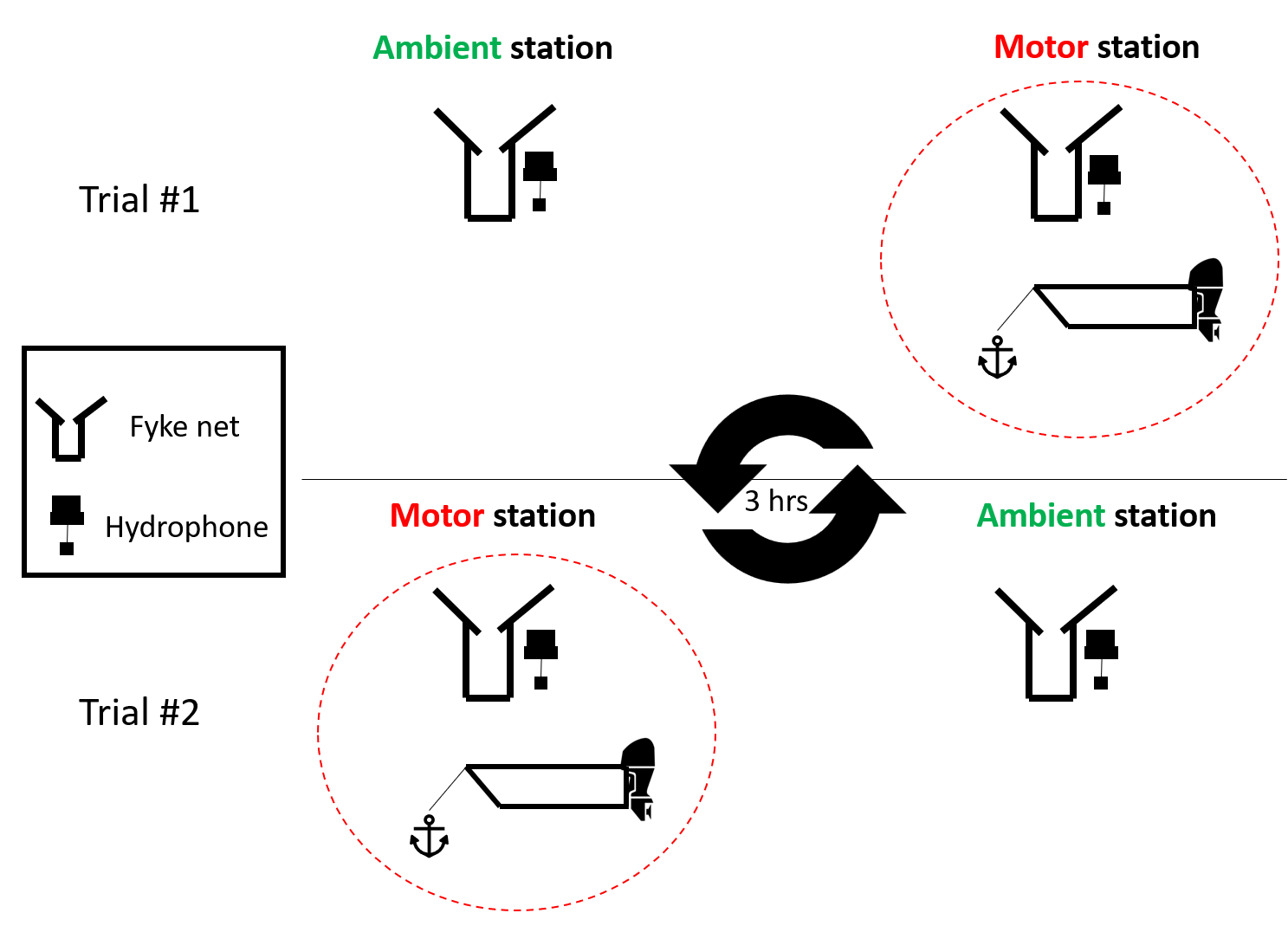
**

**Figure S2.** Experimental trap deployment sites with colors indicating the experimental pairs.


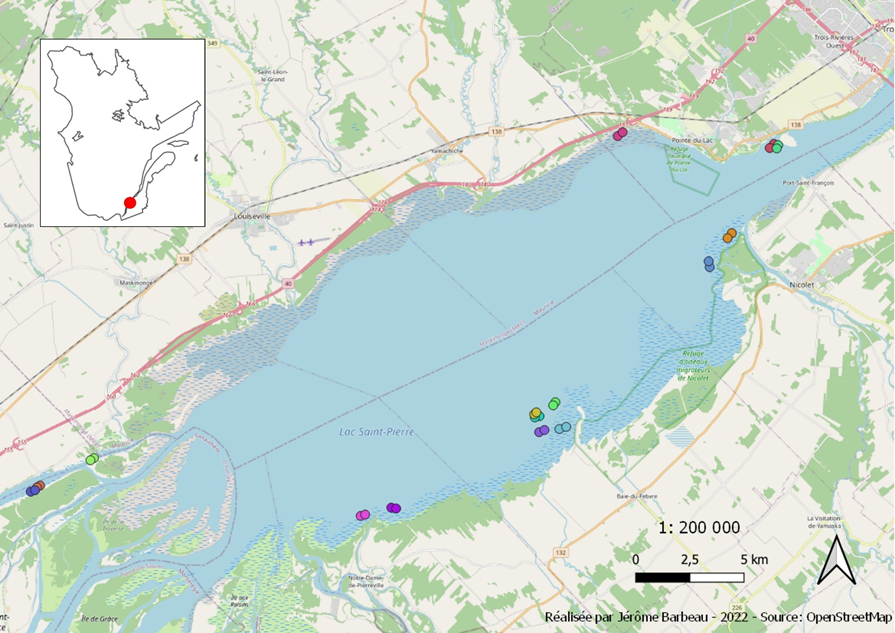


Québec

**Figure S3.** Phylogenetic relationships of the 30 species of fish studied.

**Figure S3.** Phylogenetic relationships of the 30 species of fish studied.


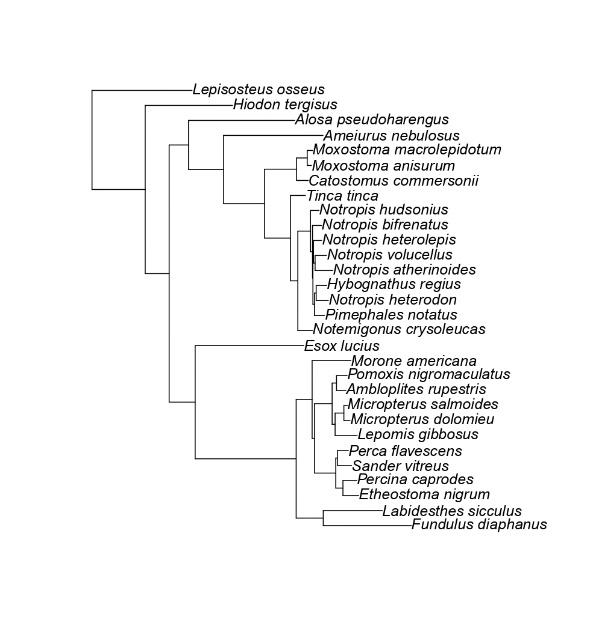


**Figure S4.** Summary of the a posteriori distribution of the regression coefficient reflecting the effect of the sound-enriched treatment on species with accessory hearing structures (mean and 95% credibility interval). The prior distribution summary is also shown (in grey).
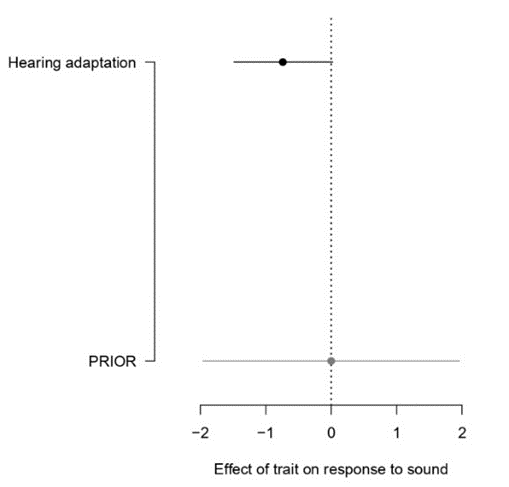

Supplement: Supplementary file 1 — Data S1 [file ECE3-14-e10946-s001.docx]
